# The Nerves to Conduct a Multiple Sclerosis Crime Investigation

**DOI:** 10.3390/ijms22052498

**Published:** 2021-03-02

**Authors:** Sameeksha Chopra, Zoë Myers, Henna Sekhon, Antoine Dufour

**Affiliations:** 1 McCaig Institute for Bone and Joint Health, Calgary, AB T2N 4N1, Canada; choprs7@mcmaster.ca (S.C.); zoemyerss@gmail.com (Z.M.); henna.sekhon@ucalgary.ca (H.S.); 2 Department of Physiology and Pharmacology, University of Calgary, Calgary, AB T2N 4N1, Canada; 3 Department of Biochemistry and Molecular Biology, University of Calgary, Calgary, AB T2N 4N1, Canada; 4 Faculty of Health Sciences, McMaster University, Hamilton, ON L8S 4L8, Canada

**Keywords:** multiple sclerosis (MS), microbiome, bacteria, virus, central nervous system (CNS), immunity

## Abstract

Multiple sclerosis (MS) is a chronic inflammatory neurodegenerative autoimmune disease characterized by the aberrant infiltration of immune cells into the central nervous system (CNS) and by the loss of myelin. Sclerotic lesions and various inhibitory factors hamper the remyelination processes within the CNS. MS patients typically experience gradual cognitive and physical disabilities as the disease progresses. The etiology of MS is still unclear and emerging evidence suggests that microbiome composition could play a much more significant role in disease pathogenesis than was initially thought. Initially believed to be isolated to the gut microenvironment, we now know that the microbiome plays a much broader role in various tissues and is essential in the development of the immune system. Here, we present some of the unexpected roles that the microbiome plays in MS and discuss approaches for the development of next-generation treatment strategies.

## 1. Introduction

Multiple sclerosis (MS) is a chronic inflammatory autoimmune disease characterized by aberrant infiltration of immune cells into the central nervous system (CNS) parenchyma [1]. Immune cell infiltration is accompanied by the loss of myelin (demyelination) and neurodegeneration of the CNS [1,2]. The accumulation of sclerotic lesions and inhibitory factors can significantly hamper remyelination processes within the CNS, causing MS patients to experience debilitating cognitive and physical disabilities as the disease progresses [1,2]. While the etiology of MS is unknown, emerging evidence continues to suggest that the microbiome may play a significant role in disease pathogenesis [1,3,4]. Autoimmune diseases, including MS, are more prevalent in countries that have higher sanitation standards and a greater use of antibiotics [5]. This phenomenon is described as the “hygiene hypothesis” [6], which suggests that overly hygienic westernized lifestyles may result in gut dysbiosis and dysregulated immune responses. Several environmental factors such as diet and vitamin D exposure have been linked to an increased risk of MS and may help explain the distinct geographical distribution of the disease [6]. People moving from countries with a low prevalence of MS to those with a high prevalence adopt a higher risk of developing the disease [6,7,8,9], perhaps facilitated by changes in lifestyle and diet that drive a shift in the composition of the intestinal flora. People with MS [10,11,12] and animal models of the disease [13] have altered gut microbiota composition, which brings us to question whether the microbiome could be a driver of MS pathogenesis. There is an imminent need to better characterize changes in the microbiome composition of individuals living with MS and study the complex mechanisms by which microbiota can influence host biology and affect disease pathogenesis (Box 1).

Box 1 **What if the CNS of MS patients were compared to a crime scene where a detective arrives to investigate the damage done and gather forensic evidence.**

**The victim: Central Nervous System**

- Damage to the myelin coating on nerves
- Evidence of an activated immune response in the CNS that limits clearance of myelin debris and prevents endogenous remyelination processes
- Microbial contributions that affect the phenotype and function of resident and peripheral immune cells

## 2. Investigating the Multiple Sclerosis Crime Scene

MS patients are monitored and diagnosed after presenting with lesions visible on an MRI that are disseminated in time and space. However, there is little known about the initiating cellular and molecular events that contribute to dysregulated inflammation prior to formation of MS lesions. There are likely drastic changes to the CNS tissue microenvironment that accompany neuroinflammation and the subsequent development of MS plaques. Many of the inhibitory factors that accumulate within plaques impair remyelination in the CNS and thus limit the organs’ endogenous tissue repair processes. 

## 3. Breaking and Entering the Blood Brain Barrier

Homeostasis is maintained in a healthy CNS through the regulated entry of substances from peripheral circulation. Tight-junction proteins create a selective blood–brain barrier (BBB) surrounding the delicate CNS tissue. However, BBB breakdown in MS patients is accompanied by increased peripheral immune cell infiltration into the CNS. Many mechanisms have been suggested as being the cause of a dysfunctional BBB, including inflammatory cytokines, activated peripheral immune cells recognizing CNS antigens, secretion of proteases and microbial derived factors [14,15]. 

Alterations in the gut microbiome could affect the permeability of the BBB and contribute to neurological diseases such as MS. In a study that investigated the effects of antibiotics on BBB integrity in a germ-free mouse model of MS, a decrease in gut microbiome diversity was positively correlated with an increase in BBB permeability and a downregulation of short-chain fatty acid (SCFA)-producing microbes [16]. Several studies have reported the effect of gut microbes and their metabolites on BBB permeability [17]. For example, germ-free mice colonized with bacterial strains that produce SCFAs reinforced the integrity of the BBB by upregulating the expression of tight-junction proteins [15]. Furthermore, resident microglial cells in the brain can also modulate BBB permeability [18]. As described next, the microbiome can influence the function of cell types resident to the CNS, highlighting the complex interplay between the gut and brain. In MS patients, a deficit of SCFA-producing bacteria likely contributes to increased BBB permeability and the subsequent entry of neuroinflammatory factors into the CNS. People with MS also often have elevated concentrations of serum zonulin [19], a protein known to be released in response to gut dysbiosis [20], which is capable of increasing intestinal permeability and breaking down the BBB through the regulation of tight-junction proteins [19,21]. Altering the microbiome or delivering BBB-reinforcing microbial metabolites may be a strategy to repair a damaged BBB, stop peripheral immune cell access into the CNS and thereby prevent the exacerbation of disease. 

## 4. Is the Peripheral Immune System an Accomplice?

**Figure 1:**
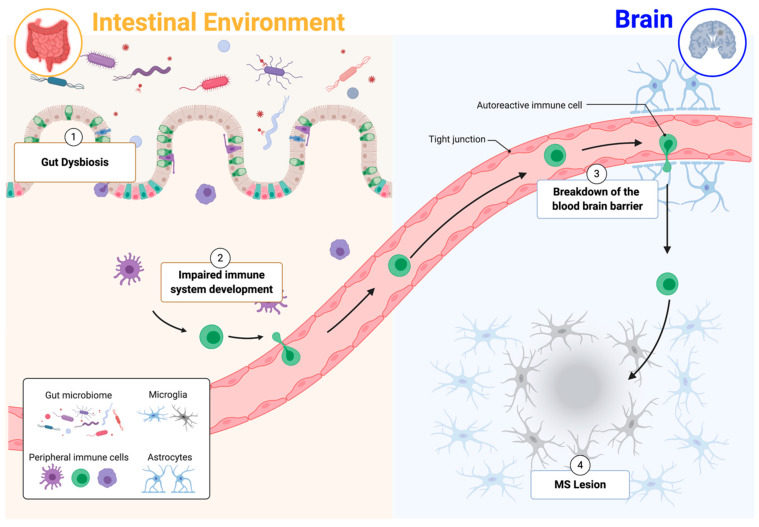
Gut–immune–brain connection. (1) Gut dysbiosis can impair mucosal immunity homeostasis. (2) Changes in immune system development are linked to multiple autoimmune diseases. (3) Blood–brain barrier breakdown is a hallmark of multiple sclerosis (MS), and the gut microbiome is a key regulator of blood–brain barrier permeability. (4) Peripheral immune–cell infiltration and subsequent autoimmune responses contribute to the development and exacerbation of MS lesions. Adapted from “Breast Cancer to Brain Metastasis”, by Biorender.com (accessed 1 March 2021). Retrieved from https://app.biorender.com/biorender-templates (accessed 2 March 2021).

The increase of immune–cell infiltration into the CNS during MS relapses supports the notion that MS is an immune-mediated disease. However, whether neuroinflammation during the early stages of MS is a result of infiltrating immune cells activated in the periphery or is a process initiated within the CNS itself remains to be characterized. Gut bacteria transferred from MS patients into mice exacerbated the development of MS-like disease in various mouse models [22,23], showing that the intestinal microbiome is capable of influencing disease progression. Despite the less obvious connection between the brain and gut, growing evidence suggests that intestinal immune responses and neuroinflammation are interconnected (Figure 1).

### 4.1. Maternal and In Utero Effects on Immune System Development

The maternal microbiome has been shown to affect fetal immune system development [24], which may confer lifelong consequences on the offspring by modulating their risk of developing autoimmune diseases such as MS. Interestingly, fetal thymic CD4+ T cell and regulatory T cell (Treg) development is compromised in germ-free mice [25]. Maternal supplementation of the intestinal bacterial metabolite acetate significantly rescued CD4+ T cell and Treg development by upregulating the autoimmune regulator (AIRE) [25], which is essential for self-tolerance induction and Treg development early in life. In humans, low maternal serum acetate levels are mirrored in the fetus, which may predispose the offspring towards impaired self-tolerance. The specific connection between the in-utero effects of bacterial-derived factors on immune development in offspring later in life, particularly in the context of multiple sclerosis, has yet to be explored in depth. A case-control study analyzed maternal vitamin D concentrations in the serum of pregnant women in the Finnish Maternity Cohort whose children had developed MS [26]. Maternal vitamin D deficiency during early pregnancy was associated with a 2-fold increase of MS risk in the offspring [26]. Low concentrations of neonatal vitamin D are also associated with increased risk of MS [27]. Cohort studies that assess bacterial metabolites using unbiased metabolomics may be insightful for understanding the contribution of the maternal microbiome in modulating the risk of an offspring developing MS. 

### 4.2. Immune System Development

Epidemiological data support the idea that exposure to environmental factors in childhood may be strong determinants of MS risk [27,28,29,30]. As migration studies suggest, the incidence rate of MS in immigrants tends to be somewhere between the incidence rate associated with their birthplace and the incidence rate associated with their final residence. If this immigration happens in childhood, then the incidence rate tends to be closer to that associated with their final residence [27,30,31]. For example, natives of the Caribbean islands and Asia do not experience a significantly increased risk of developing MS upon immigrating to the United Kingdom (UK); however, high rates of MS are documented in their U.K.-born children [30]. This may suggest that individuals who spend their early life in low-risk areas tend to benefit from long-lasting protection against MS that is not transferred to their children [27,30], hinting at the possibility that these factors may include bacteria and viruses that shape the development of their immune system. Perhaps there are vulnerable periods early in one’s life where environmental factors are particularly critical for immune system development and can affect one’s future risk of developing MS or other autoimmune diseases.

### 4.3. Gut Microbiome Development

The gut microbiome plays an essential role in the development of the immune system [32]. Gut microbes and the host exist in a symbiotic relationship and when this relationship becomes dysregulated, dysbiosis can occur, resulting in defects in the immune response, immune system disorders and triggering of autoimmune diseases. During fetal and infant development, the gut microbiome develops alongside the immune system [33]. As demonstrated in germ-free mice, the gut must be colonized with microbes in order for the immune system to properly mature. Initial colonization of gut microbes occurs *in utero*, indicating a possible transfer of bacteria from the blood stream of the mother to the fetus, which continues after birth as the infant breastfeeds and begins eating solid food [34]. The method of delivery may affect the development of the gut microbiota as it is suggested that Caesarian-delivered babies have low bacterial diversity within their gut [34]. These infants are more susceptible to immune disorders like asthma and Celiac disease [34,35]. Similar results were seen in infants who were fed baby formula rather than breast milk, indicating that there are various environmental factors that play a role in the development of the gut microbiome and that a slightly impaired gut microbiota (decrease in diversity) may lead to immune disorders later in life. Additionally, the immunogenicity of early colonizing intestinal bacteria may have profound impacts on the repertoire of immune responses later in life. Vatanen et al. followed gut microbiome development in 222 infants in Northern Europe from birth until age three [36]. Bacteroides species that inhibit innate immune signalling were lowly abundant in infants from Russia, where early-onset autoimmune diseases are less prevalent, compared to Finland and Estonia [36]. In line with the “hygiene hypothesis”, early colonization with immunologically silencing microbiota may impair immune education and maturation. 

### 4.4. Short-Chain Fatty Acids (SCFAs) Can Modulate Immunity

Gut bacteria play a crucial role in extracting key nutritional factors from a healthy diet. Non-digestible carbohydrates can be fermented by specific bacterial species to make metabolites that modulate host biology [37,38]. SCFAs produced by the intestinal microbiota are critical for the development and maintenance of a healthy immune system [37,39]. Importantly, SCFAs like acetate, propionate, butyrate and pentanoate, can exert anti-inflammatory effects on the host and are often downregulated in chronic inflammatory conditions [40]. In MS patients, serum concentrations of butyric acid (BA) are reduced and medium-chain fatty acids (MCFAs), such as caproic acid (CA), are increased [41]. The altered BA/CA ratio correlates with the immunological profile of MS patients, as they present with an increase in Th1 and Th17 cells as well as a decrease in Treg lymphocytes [41]. Concurrently, the microbiota of MS patients is depleted of BA producers [41]. Decreased serum butyric acid levels correlate with increased intestinal permeability as measured by serum intestinal fatty acid binding protein (I-FABP) and bacterial lipopolysaccharide (LPS) in MS patients compared to healthy controls. The presence of these factors in the blood suggests impaired gut barrier integrity in MS. Pentanoate is a potent regulator of immunometabolism as it can reprogram the metabolic activity of lymphocytes. In experimental mouse models of multiple sclerosis, pentanoate-induced regulatory B cells protect against autoimmunity [42]. Additionally, pentanoate induces IL-10 and reduces IL-17A production by epigenetically modifying CD4 T cells [42]. Germ-free mice are highly resistant to the development of experimental autoimmune encephalomyelitis (EAE), a mouse model of MS. However, segmented filamentous bacteria (SFB) induce pathogenic Th17 immune responses and promote CNS inflammation in SFB mono-colonized germ-free animals [42]. Interestingly, administering pentanoate in SFB-colonized germ-free mice ameliorates EAE and decreases Th17 cells in the CNS [42]. 

## 5. The Gut, an Unexpected Suspect in MS, Modifies the Autoimmune Response

### 5.1. Macrophages and Microglia

Microglia are tissue-resident immune cells in the CNS that can influence inflammation and neurodegeneration by secreting factors and coordinating with other cell types, such as astrocytes, oligodendrocytes, neurons and peripheral immune cells that have infiltrated into the CNS [43]. A thorough characterization of how the microbiota influences the function of neural cell types in MS remains to be done. Gut microbiota can metabolize dietary tryptophan to produce aryl hydrocarbon receptor (AHR) agonists that can limit CNS inflammation by acting on AHR receptors expressed by microglial cells [44]. Tryptophan metabolism has been of interest in autoimmunity research because metabolic products of the kynurenine pathway, which are used to metabolize tryptophan, are known to exert several effects on the immune system, including modulating immunotolerance [45]. Several studies have investigated the levels of kynurenines in MS patients or in the EAE mouse model, demonstrating that aberrant kynurenine pathway activity is associated with increased severity of disease [45,46,47,48]. Tryptophan-derived metabolites, such as indole-3-propinonic acid, are produced by the microbiota and are associated with MS relapses [45]. However, further research is required to understand the specific pathophysiological role of tryptophan-derived microbial metabolites in MS. Bacterial products, such as SCFAs, from members of the gut microbiota play a substantial role in microglia homeostasis as temporal eradication of the host microbiota or limited microbial complexity can significantly change microglia properties [49]. Duscha et al. showed that supplementing MS patients with the SCFA propionic acid increases Tregs and can be a promising immunomodulatory supplement to MS drugs [50]. While the study did not assess the impact of SCFAs on tissue resident immune cells in the CNS, it is likely that SCFA treatment modulates the maturation and activation of microglia as well [49]. Ultimately, applying our understanding of microbiome–host interactions specifically pertaining to the CNS may help to design better therapeutic approaches for neurological illnesses. 

### 5.2. T Cells

The gut–brain axis plays a key role in neuroimmunology, and emerging evidence suggests that encephalitogenic immune responses may be driven by intestinal dysbiosis in MS [51,52]. Th17 cells are key mediators of CNS autoimmunity and their role in MS has been established in both humans and in animal models [53]. Specific microbiota modifications in the human intestinal microenvironment, including a higher *Firmicutes*/*Bacteroidetes* ratio, increased relative abundance of *Streptococcus*, and decreased *Prevotella* strains, have been implicated in promoting Th17 cell expansion and correlating with brain autoimmunity in MS patients with high disease activity [51]. In an adoptive-transfer mouse model of MS, CNS-specific Th17 cells first migrate to the intestine to proliferate during disease pathogenesis prior to reaching the CNS and inducing neurological symptoms [53]. The pro-inflammatory properties of encephalitogenic Th17 cells are strengthened in part by changes in gut microbiota. Blocking α4β7-integrin and its cognate ligand mucosal addressin cell adhesion molecule 1 (MAdCAM-1) disrupts Th17 cell intestinal homing and attenuates EAE severity [53,54]. In another study, eradication of the gut microbiome using antibiotics prevented the induction of CNS inflammation by gut-derived T cells and abolished the development of EAE [55]. These studies implicate the intestine as a checkpoint for regulating autoimmune T cells. The gut microbiota may also modulate the disease course of MS. In the EAE model, differentially abundant intestinal bacteria determine susceptibility to chronic-progressive versus relapse-remitting forms of disease [56]. The contribution of intestinal T cell subsets, including the presence of mucosal-associated invariant T (MAIT) cells in brain lesions [57] and peripheral circulation of MS patients [58], is starting to be recognized in the pathophysiology of MS. Ultimately, studies in animal models and patient data suggest that the microbial composition of the gut may play a critical role in catalyzing CNS-specific autoimmune responses in MS.

### 5.3. B Cells

Gut-associated B cells, most of which differentiate into IgA-secreting plasma cells (PCs), are predominantly found in Peyer’s patches [59]. IgA antibodies are a critical first line of defence against antigens in the intestinal microenvironment. During EAE, there is a downregulation of IgA PCs in the gut and an upregulation of IgA PCs in the CNS [60]. PCs that egress from the gut into the CNS suppress the symptoms of EAE via the interleukin (IL)-10 pathway [60]. Therefore, intestinally derived IgA B cells may represent a population of regulatory cells that can be recruited to tissues to mediate inflammation independent of their receptor specificities. Also, intestinal PCs may be neuroprotective because they migrate to, and limit the neuroinflammation of, the damaged CNS. Future studies should test the efficacy of treatments that mobilize IgA PCs in MS. Overall, the presence and the functions of immunosuppressive IgA PCs in the CNS of MS patients needs to be further characterized.

## 6. Immune Activation: What Is the Motive for the Crime?

Autoimmune responses often exacerbate MS pathogenesis; however, the specific triggers that initiate the activation of pathogenic self-reactive immune cells that lead to the onset of MS remain unidentified. One possible trigger is the cross-reactivity of immune cells to microbiota-derived peptides [61]. Genetic and environmental risk factors are likely both implicated in MS pathogenesis [2]. Some of the most common environmental risk factors are viral infections [2] (Table 1). Viral models of MS, such as the Theiler’s murine encephalomyelitis virus (TMEV) model [62], are commonly used to study MS pathology. The need to study viral contributions to disease is emerging because pathogenic human endogenous retroviruses (HERVs), normally dormant and comprising 8% of the human genome, are upregulated in response to environmental factors in MS [63,64,65]. Another virus implicated in MS pathogenesis is the Epstein–Barr virus (EBV), a member of the herpes virus family that causes infectious mononucleosis [66,67]. While more than 95% of healthy individuals demonstrate an immune response to EBV, virtually all MS patients are seropositive for EBV [68,69]. Libbey et al. [70] hypothesize that MS could be triggered via molecular mimicry, whereby specific peptides secreted by pathogens have sequence or structural similarities to self-antigens. Previous studies have suggested the role of molecular mimicry in the pathogenesis of MS through Haemophilus influenzae viral infections [71], and a potentially similar mechanism has been observed for EBV infection [72]. The proposed cascade of events leading to CNS autoimmunity following EBV infection involves the proliferation and maturation of T-cells against Epstein–-Barr virus nuclear antigen 1 (EBNA1) [73]. Tengvall et al. [73] hypothesized that T cell responses against EBNA1 may cross-react with a protein known as anoctamin 2 (ANO2), which is expressed in the CNS. Interestingly, autoantibody levels against ANO2 have been shown to be increased in the cerebral spinal fluid (CSF) of MS patients [73]. ANO2 is a calcium-activated chloride channel important for ion transport and control of neuronal excitability that is expressed in the hippocampal and cortical regions and specifically detected near and inside MS plaques [73]. EBV remains a *priority suspect* because (1) MS is potentially closely linked with an immune response to EBV, and (2) a history of mononucleosis doubles the risk for MS [73]. Molecular mimicry remains the primary explanation for the link between EBV and MS. 

Other viruses have also been implicated in the pathogenesis of MS. Human herpesvirus 6 (HHV6) is a member of the herpesvirus family and has two subtypes: HHV6A and HHV6B [74]. Both types preferentially attack or affect the nervous system, but HHV6A has greater neurotropism, which may imply a closer link to MS [74]. Opsahl and Kennedy detected significantly higher levels of HHV6 in post-mortem lesioned brain tissue from MS patients compared to that of healthy controls [75]. Another study reported that a high antibody response against the HHV6A-specific antigen immediate-early protein 1A (IE1A) positively correlates with MS [76]. Individuals with strong humoral immunity against IE1A, particularly those younger than 20 years old, have a higher risk of developing MS later in life [76]. The positive association between the IE1A antigen from HHV6-A and MS is intriguing, considering that HHV6A can impede the migration of oligodendrocyte progenitor cells and establish latent infection in oligodendrocytes [77], the myelin-producing cells presumed to be a target of the MS-related autoimmune responses. However, the mechanistic role of HHV6 in the pathology of the disease remains unknown. Human endogenous retroviruses (HERVs) are usually epigenetically silenced within our genome but certain triggers, including transactivation by exogenous viral infections such as EBV, may lead to the re-expression of HERVs. HERV type W (HERV-W), formerly known as multiple sclerosis-associated retrovirus (MSRV), was discovered in the CSF of MS patients and is frequently detected in the brains of MS patients [78]. In MS lesions, myeloid cells contain the envelope protein ENV from HERV-W, which has recently been shown to polarize microglia and contribute to neurodegeneration [78]. A Phase IIb clinical trial (NCT02782858) tested the efficacy of GNbAC1 treatment, a humanized anti-ENV IgG_4_ monoclonal antibody [79]. GNbAC1 exerted significant neuroprotective effects in MS patients within 1 year of treatment, including (1) a 31% reduction in cortical atrophy, (2) 72% reduction in thalamic atrophy and (3) 63% reduction of T1 hypointense lesions correlating with permanent brain tissue damage [78,79]. Neurotropic polyomaviruses (PyV) such as JCPyV and Simian Virus 40 (SV40) have also been associated with MS [80]. Significantly lower levels of IgG antibodies against JCPyC viral capsid protein antigen 1 (VP1) epitopes were detected in MS patients compared to healthy controls [80]. Similar outcomes were observed using an indirect enzyme-linked immunosorbent assay (ELISA) with two synthetic peptides mimicking SV40 antigens [81]. MS patients had a significantly lower prevalence of SV40 antibodies but a higher prevalence of soluble HLA-G (sHLA-G) molecules as compared to healthy controls, non-MS inflammatory diseases or non-inflammatory neurological diseases [80,81,82]. These studies suggest a general trend of MS patients having a diminished ability to counteract JCPyV or SV40 infections via antibody production. There is likely an association between viral infections and MS; however, there is still a need to determine if there is a causal relationship between viruses and the onset of MS. 

Another area of MS research that remains underexplored is the contribution of bacteriophages, viruses that infect bacteria. It is plausible that bacteriophages are the underlying cause of intestinal dysbiosis in MS patients, but they have also been implicated in the mimicry of MS-relevant autoantigens. The *Synechococcus* phage is suggested as a major contributor to the molecular mimicry phenomenon [83], whereby antibodies raised to viral proteins cross-react with self-antigens leading to autoimmune responses. Bacteriophages may also induce chronic inflammation by changing gut microbiome composition and in turn increasing intestinal permeability [84,85]. Zhao et al. found less diversity in the intestinal virome of children who subsequently developed serum autoantibodies associated with progression to type 1 diabetes (cases) as compared to healthy controls [86]. Populations of bacteriophages differed significantly between cases and controls [86]. The disease-discriminating bacteriophage sequences identified also significantly correlated with different bacterial OTUs in both cases and controls, suggesting that unique virome–bacterial microbiome interactions were present prior to the development of autoantibodies [86]. Ultimately, epidemiological evidence, genetic association and mechanistic data in humans is needed to elucidate the role of viruses in the pathogenesis of MS. To this end, a broader characterization of the MS patient virome [87] will be valuable. 

## 7. Deception in the Interrogation Room: Crosstalk between Host and Bacteria

The investigation of the complex crosstalk between host and bacteria is still in its infancy (Table 1). Bacteria-derived factors have been shown to influence host proteases [88], and host proteins were shown to modulate the activity of bacterial proteases [89]. In MS patients, the human protease inhibitor, cystatin C, is significantly upregulated in brain biopsies [90]. A bacterial protease, IdeS, produced by the human pathogen *Streptococcus pyogenes* (Group A Streptococcus [GAS]) hijacks mechanisms deployed by the host to inhibit proteases by using cystatin C as a cofactor to increase its own activity [89]. The intriguing finding that a host protease inhibitor may increase bacterial protease activity further supports our lack of understanding of the complexity of host-microbial interactions. Cystatin C is also highly expressed in the brains of EAE mice [90]. Whether it interacts with bacterial proteases to influence disease pathogenesis in MS is currently unknown; however, it does seem to have a detrimental function in myelin oligodendrocyte glycoprotein 35–55-induced EAE in female mice but not in males, thus demonstrating a sex-specific role [90]. An analysis of the fecal proteome of EAE mice during disease latency demonstrated a transient increase in host protease inhibitors that inversely correlated with disease severity [91]. The administration of the antibiotic vancomycin attenuated EAE symptoms and elevated protease inhibitors [91], which further supports the idea that the microbiota modulates host biology.

**Figure 2:**
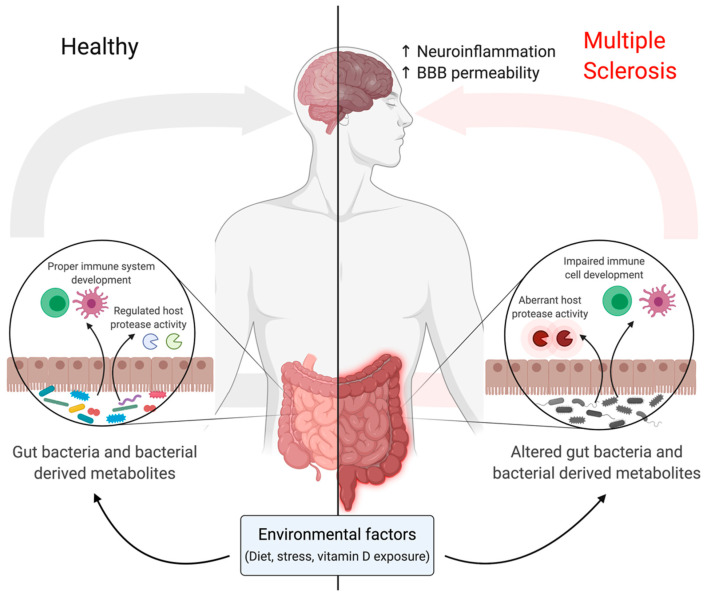
Gut microbiome can modulate host biology. Known environmental factors such as diet, stress and vitamin D levels can exacerbate MS by impairing immune system development and maturation, failing to regulate host protease activity and increasing the permeability of the blood brain barrier (BBB). Created with BioRender.com (accessed on 15 December 2020).

Guo et al. [88] identified microbial metabolites with protease inhibitory activity produced by the gut bacteria of most healthy people. These metabolites affect the activity of cathepsins in human cell proteomes [88], thereby demonstrating new biological roles in interspecies signalling. Compared to healthy controls, MS patients present with a significant decrease in the abundance of certain bacteria, including species belonging to the genus *Clostridium* [92], which may produce microbial metabolites that inhibit host cell cathepsins [88]. Accordingly, MS patients have increased cathepsin activity, which may contribute to disease pathogenesis (Figure 2). Hypomethylation of the cathepsin Z locus and increased cathepsin Z transcripts were detected in the pathology-free regions of MS brains as compared to healthy controls [93]. EAE mice deficient in cathepsin Z exhibit attenuated neuroinflammation and demyelination [94]. Cathepsin Z deficiency dramatically reduced circulating interleukin 1 beta levels and compromised the ability of mice to mount Th17 responses critical for the development of EAE [94]. Perhaps, restoring balance to the gut microbiome may help to regulate the aberrant activity of host proteases such as cathepsins seen in chronic inflammatory diseases such as MS. 

Metabolites produced by gut bacteria are also capable of influencing immune homeostasis [95]; therefore, alterations in gut bacteria composition may have drastic effects on immune responses (Figure 2). CD4^+^Foxp3^+^ Tregs are critical mediators of immune homeostasis and the development of this cell population is directly affected by interactions with gut microbiota [95]. The spore-forming components of intestinal microbiota, specifically *Clostridia* clusters IV and XIVa, was shown to promote Tregs by providing an environment rich in transforming growth factor (TGF)-β to drive Treg development [96]. The microbiome of MS patients has a reduction of bacteria belonging to *Clostridia* clusters IV and XIVa, potent producers of SCFAs, which regulate inflammation [92,97]. Mizuno et al. [97] demonstrated that administration of SCFAs suppresses lymphocyte-mediated systemic inflammation in EAE, thereby reducing EAE severity. Ultimately, further research characterizing changes in bacteria–host interactions will contribute to a stronger understanding of the molecular mechanisms affecting MS pathogenesis.

## 8. Interrogating the Suspect: Is the Gut behind the Increased Crime Rate in the CNS?

Animal models of MS have provided novel insights into the effect of the gut microbiota on MS pathogenesis. In the EAE murine model of MS, perturbing the gut microbiota has been shown to affect disease susceptibility via modulation of immune responses [98]. Interestingly, germ-free (GF) mice are resistant to the development of EAE, and show attenuated signs of disease, reduced clinical scores and shorter duration of EAE symptoms [99], which is likely a result of broadly impaired immune system development in mice that lack an intestinal microbiome [100]. While the molecular interactions between intestinal microbial organisms and the host were shown to affect the balance between pro- and anti-inflammatory immune responses, the immune system in return may further affect the composition of the microbiota [101]. However, changing the gut microbiome composition through the administration of probiotics does not significantly improve remyelination potential in mice afflicted with EAE [101]. Additional research is needed to delineate the cause-and-effect relationship between the role of the microbiome in MS. In cancer immunotherapy, intestinal bacteria have been associated with enhanced efficacy of immune checkpoint inhibitors [102]. Future studies should consider assessing the efficacy of probiotic supplementation in conjunction with pharmacological inhibitors and lifestyle changes to elucidate potential synergistic effects of probiotic therapy on maximizing benefit afforded to people living with MS. The human gut commensal *Prevotella histicola* (*P. histicola*) was demonstrated to be as effective as the immunomodulatory drug glatiramer acetate at suppressing disease severity in a preclinical mouse model of MS [103]. While a combination of *P. histicola* and glatiramer acetate did not result in an additive effect on reduced EAE disease severity [103], it would be interesting to investigate whether *P. histicola* works synergistically with a different pharmacological drug approved for MS. 

## 9. Profiling Other Tissues beyond the Gut

While the gut has been implicated in the initial priming and proliferation of autoreactive CNS-specific immune cells, the lungs are another site potentially important for the reactivation of immune cells prior to disease relapse [104]. MS patients have elevated breath methane [10] which is often attributed to the presence of methanogens such as *Methanobrevibacter* in the gut. Methanogens are also found in the respiratory tract [105,106]; however, the lung microbiome is currently undercharacterized in health and disease. To better characterize the role that the microbiota plays in MS, it is important to study the microbiome of MS patients at sites other than the gut, such as the microbiota of the skin and lungs. Besides altered intestinal bacteria composition in chronic inflammatory diseases, the influence of other environmental factors––like changes in the virome, fungome or parasitome––remain underexplored. Importantly, certain microbes and their products have been implicated in exerting protective effects in MS [107,108,109]. The infection of MS patients or animal models of the disease with helminth parasites has been shown to be protective due to beneficial immune modulation and improvements in clinical symptoms [107]. Unbiased Omics technologies––like genomics, transcriptomics, proteomics and metabolomics––are valuable tools for enhancing our understanding of the human microbiome as a whole [110]. 

## 10. Chasing a Cure: MedXercise

**Figure 3:**
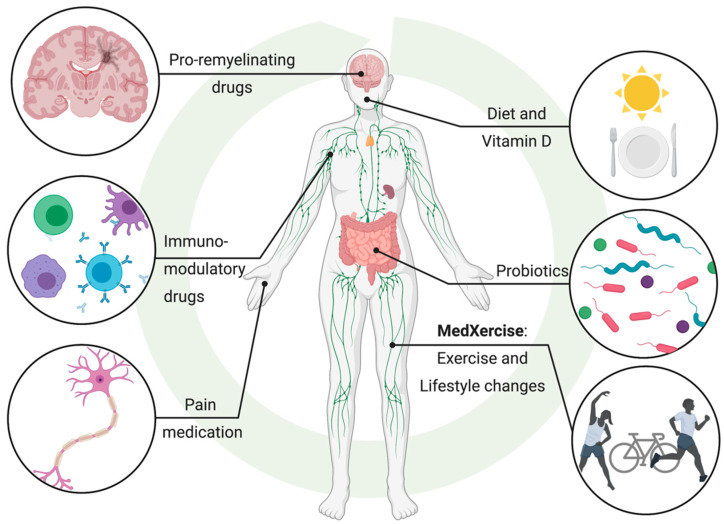
Multimodal approach to managing and treating MS and other chronic inflammatory diseases. Created with BioRender.com (accessed on 26 January 2021).

Exercise is known to exert a wide range of health benefits. A review by Lozinski and Yong presented the impact of exercise on structural and functional changes in the CNS [111]. The therapeutic effects of exercise in MS may, in part, be mediated by direct immunomodulatory effects since exercise is known to promote anti-inflammatory immune responses [112]. However, exercise may also induce beneficial changes for patients with neuroimmune illnesses by shifting the composition of their microbiota [113,114,115,116,117,118]. Whether these effects are solely attributable to physical activity or are influenced by other factors associated with healthier lifestyle, such as an altered diet, needs to be investigated [119]. It is important to note that the benefits of exercise may be dependent on the composition of the microbiota [120]; thus, combined treatment targeting the microbiota and engaging in exercise may maximize therapeutic effects (Figure 3). Interestingly, a physically active lifestyle is associated with beneficial changes to the microbiome [121] and it may prove to be useful in managing MS. Exercise has been shown to work in combination with pharmacological interventions such as clemastine [122], also known as meclastin, a drug that promotes remyelination and is a H1 histamine antagonist. In a toxin-induced model of focal demyelination, exercise promotes changes in the lesion microenvironment and makes the CNS more conducive to repair [122]. Exercise was shown to be as equally efficacious as the pharmacological treatment clemastine at promoting remyelination of lysolecithin-induced lesions in mice [122]. Furthermore, exercise and clemastine used in combination work synergistically to additively enhance remyelination [122]. While exercise exerts direct neuroprotective effects by promoting anti-inflammatory innate immune responses and increasing phagocytosis of myelin debris [122], it would be interesting to explore whether these changes are correlated with a shift in the microbiome. Nonetheless, the potential to couple pharmacological interventions with an adjunctive therapy like exercise, a concept termed MedXercise [111], is exciting because it provides a new means by which the maximal treatment benefit can be afforded. 

## 11. The Evidence Speaks: Multifactorial Diseases Must Be Treated by Multimodal Means

For a complex disease like MS, individual drugs have proven to be ineffective for management and treatment and have failed to inhibit MS progression. A multimodal approach may be more effective at addressing complex chronic diseases, including MS and its comorbidities [123]. Administering a pro-remyelinating drug may not be as efficacious for long-term use without reducing recurring chronic inflammation. Importantly, the immunomodulatory roles of the microbiota and other environmental or lifestyle factors should be considered. McMurran et al. showed that inflammatory responses during remyelination depend on the composition of the microbiota, but interventions that modify the microbiota have a minimal impact on endogenous CNS remyelination [101]. Here, a multimodal approach to modulating the microbiota along with taking a pro-remyelinating drug is an optimal treatment strategy, as shown by Jensen and colleagues [122]. Additionally, monoclonal antibodies, such as natalizumab, which is approved for MS [124,125], have been shown to be efficacious at preventing leukocyte trafficking into the CNS [126], but whether this therapy can be combined with exercise or other means to modulate the microbiome to reduce inflammatory immune cells warrants further investigation. While pharmacological treatment may focus on one aspect of a disease, an effective “cure” will need to address all dysregulated features of MS. It is likely that microbiota composition plays an influential role in MS pathogenesis. Correcting intestinal dysbiosis may also improve cognitive behaviour and depression [127], which is a common symptom of MS [123,128]. Therefore, future treatments should consider how disease-modifying treatments (DMTs) can be combined with therapies targeted at the microbiota. These include lifestyle changes [129] such as exercise and modified diets, to maximize tissue repair and clinical benefit for MS patients (Figure 3). This may be of particular importance for MS patients residing in long-term care facilities as nursing home residents’ microbiomes are associated with decreased SCFA-producing organisms and increased intestinal dysbiosis [130], which may exacerbate MS pathogenesis. Multicenter studies across the world may help identify microbial strains common to MS patients independent of the vastly different lifestyles and diets of patients from different countries. However, it is important to consider the effects of treatment and disease duration on the microbiome of MS patients, as several current or promising DMTs––glatiramer acetate [131], minocycline [132], fingolimod [133], teriflunomide [134] and dimethyl fumarate [134]––have antimicrobial effects. Another critical point is the importance of characterizing and subcategorizing various phenotypes and clinical parameters, such as disease progression, which may influence the microbiome of MS patients. It is likely that “the crime of MS” is committed by more than one criminal. Therefore, solving the cold case of MS will require the combination of multiple experts from various disciplines including microbiology, virology, neurology and immunology. 

## Figures and Tables

**Table 1:** List of suspects involved in multiple sclerosis.

Organism	Suggested Mode of Action	Reference
Short-chain fatty acid (SCFA)-producing bacteria	Downregulation leads to increased BBB permeability	[16]
	Supplementation in germ-free mice reinforces BBB integrity by upregulating tight-junction proteins	[15]
	Maternal supplementation with the SCFA acetate supports fetal CD4 T cell and regulatory T cell development by increasing AIRE	[25]
	Decreased SCFA butyric acid and increased MCFA caproic acid levels correlate with increased Th1, increased Th17 and decreased regulatory T cells in MS patients	[41]
	SCFA pentanoate induces regulatory B cells, increases IL10 and reduces IL17A by epigenetically changing CD4 T cells in EAE	[42]
	Supplementing MS patients with SCFA propionic acid increases regulatory T cells	[50]
Bacteroides species	Inhibits innate immune signalling; in low abundance in infants from Russia, where early-onset autoimmune diseases are less prevalent	[36]
Segmented filamentous bacteria (SFB)	Induce pathogenic Th17 imune responses; increased CNS inflammation in SFB-monocolonized animals	[42]
Streptococcus strains	Increased levels promotes Th17 cell expansion and correlates with brain autoimmunity in MS patients with high disease activity	[51]
Prevotella strains	Decreased levels correlate with increased brain autoimmunity in MS patients with high disease activity	[51]
	*Prevotella Histicola* administration is as effective as glatiramer acetate at suppressing EAE disease activity	[103]
Clostridia clusters IV and XIVA	Promote regulatory T cells; potent producers of SCFAs	[92,96,97]
Epstein–Barr Virus (EBV)	Molecular mimicry between viral EBNA1 protein and human calcium activated chloride channel Anocatamin 2 (ANO2)	[72,73]
Human Herpesvirus 6A (HHV6)	Neurotropic virus; increased HHV6 levels in post-mortem lesioned brain tissue from MS patients as compared to healthy controls	[74,75]
	Increased seroreactivity against HHV6A specific antigen immediate early protein 1A (IE1A) positively correlates with MS	[76]
	HHV6A impedes oligodendrocyte precursor cell migration and establishes latent infection in oligodendrocytes	[77]
Human endogenous retrovirus type W (HERV-W)/MS associated retrovirus (MSRV)	Frequently detected in MS patient brains; HERV-W envelope protein ENV polarizes microglia and contributes to neurodegeneration	[78]
	Phase IIb clinical trial of GNbAC1, an anti-ENV IgG4 monoclonal antibody, shows neuroprotective effects	[79]
Polyomaviruses (PyV)	Lower prevalence of serum antibodies against JCPyC and Simian virus 40 (SV40) in MS patients; may suggest that MS patients have diminished ability to counteract PyV infections via antibody production	[80,81,82]
Helminth parasites	Potentially protective in MS through immunomodulation and improving clinical symptoms	[107]

## Data Availability

Not applicable.

## References

[1] Filippi M., Bar-Or A., Piehl F., Preziosa P., Solari A., Vukusic S., Rocca M.A. (2018). Multiple sclerosis. Nat. Rev. Dis. Primers.

[2] Dendrou C.A., Fugger L., Friese M.A. (2015). Immunopathology of multiple sclerosis. Nat. Rev. Immunol..

[3] Filyk H.A., Osborne L.C. (2016). The Multibiome: The Intestinal Ecosystem’s Influence on Immune Homeostasis, Health, and Disease. EBioMedicine.

[4] Cryan J.F., O’Riordan K.J., Sandhu K., Peterson V., Dinan T.G. (2020). The gut microbiome in neurological disorders. Lancet Neurol..

[5] Okada H., Kuhn C., Feillet H., Bach J.-F. (2010). The ‘hygiene hypothesis’ for autoimmune and allergic diseases: An update: The ‘hygiene hypothesis’: An update. Clin. Exp. Immunol..

[6] Bach J.-F. (2018). The hygiene hypothesis in autoimmunity: The role of pathogens and commensals. Nat. Rev. Immunol..

[7] Ahlgren C., Lycke J., Odén A., Andersen O. (2010). High risk of MS in Iranian immigrants in Gothenburg, Sweden. Mult. Scler..

[8] Wallin M.T., Page W.F., Kurtzke J.F. (2009). Migration and multiple sclerosis in Alaskan military veterans. J. Neurol..

[9] Guimond C., Dyment D.A., Ramagopalan S.V., Giovannoni G., Criscuoli M., Yee I.M., Ebers G.C., Sadovnick A.D. (2010). Prevalence of MS in Iranian Immigrants to British Columbia, Canada. J. Neurol..

[10] Jangi S., Gandhi R., Cox L.M., Li N., von Glehn F., Yan R., Patel B., Mazzola M.A., Liu S., Glanz B.L. (2016). Alterations of the human gut microbiome in multiple sclerosis. Nat. Commun..

[11] Chen J., Chia N., Kalari K.R., Yao J.Z., Novotna M., Paz Soldan M.M., Luckey D.H., Marietta E.V., Jeraldo P.R., Chen X. (2016). Multiple sclerosis patients have a distinct gut microbiota compared to healthy controls. Sci. Rep..

[12] Ventura R.E., Iizumi T., Battaglia T., Liu M., Perez-Perez G.I., Herbert J., Blaser M.J. (2019). Gut microbiome of treatment-naïve MS patients of different ethnicities early in disease course. Sci. Rep..

[13] Johanson D.M., Goertz J.E., Marin I.A., Costello J., Overall C.C., Gaultier A. (2020). Experimental autoimmune encephalomyelitis is associated with changes of the microbiota composition in the gastrointestinal tract. Sci. Rep..

[14] Almutairi M.M.A., Gong C., Xu Y.G., Chang Y., Shi H. (2016). Factors controlling permeability of the blood–brain barrier. Cell Mol. Life Sci..

[15] Braniste V., Al-Asmakh M., Kowal C., Anuar F., Abbaspour A., Toth M., Korecka A., Bakocevic N., Ng L.G., Kundu P. (2014). The gut microbiota influences blood-brain barrier permeability in mice. Sci. Transl. Med..

[16] Wu Q., Zhang Y., Zhang Y., Xia C., Lai Q., Dong Z., Kuang W., Yang C., Su D., Li H. (2020). Potential effects of antibiotic-induced gut microbiome alteration on blood–brain barrier permeability compromise in rhesus monkeys. Ann. N. Y. Acad Sci..

[17] Parker A., Fonseca S., Carding S.R. (2020). Gut microbes and metabolites as modulators of blood-brain barrier integrity and brain health. Gut Microbes.

[18] Haruwaka K., Ikegami A., Tachibana Y., Ohno N., Konishi H., Hashimoto A., Matsumoto M., Kato D., Ono R., Kiyama H. (2019). Dual microglia effects on blood brain barrier permeability induced by systemic inflammation. Nat. Commun..

[19] Camara-Lemarroy C.R., Silva C., Greenfield J., Liu W.-Q., Metz L.M., Yong V.W. (2020). Biomarkers of intestinal barrier function in multiple sclerosis are associated with disease activity. Mult. Scler..

[20] Fasano A. (2020). All disease begins in the (leaky) gut: Role of zonulin-mediated gut permeability in the pathogenesis of some chronic inflammatory diseases. F1000Res.

[21] Rahman M.T., Ghosh C., Hossain M., Linfield D., Rezaee F., Janigro D., Marchi N., van Boxel-Dezaire A.H.H. (2018). IFN-γ, IL-17A, or zonulin rapidly increase the permeability of the blood-brain and small intestinal epithelial barriers: Relevance for neuro-inflammatory diseases. Biochem. Biophys. Res. Commun..

[22] Berer K., Gerdes L.A., Cekanaviciute E., Jia X., Xiao L., Xia Z., Liu C., Klotz L., Stauffer U., Baranzini S.E. (2017). Gut microbiota from multiple sclerosis patients enables spontaneous autoimmune encephalomyelitis in mice. Proc. Natl. Acad. Sci. USA.

[23] Cekanaviciute E., Yoo B.B., Runia T.F., Debelius J.W., Singh S., Nelson C.A., Kanner R., Bencosme Y., Lee Y.K., Hauser S.L. (2017). Gut bacteria from multiple sclerosis patients modulate human T cells and exacerbate symptoms in mouse models. Proc. Natl. Acad. Sci. USA.

[24] Gomez de Aguero M., Ganal-Vonarburg S.C., Fuhrer T., Rupp S., Uchimura Y., Li H., Steinert A., Heikenwalder M., Sauer U., McCoy K.D. (2016). The maternal microbiota drives early postnatal innate immune development. Science.

[25] Hu M., Eviston D., Hsu P., Mariño E., Chidgey A., Santner-Nanan B., Wong K., Richards J.L., Yap Y.A., Collier F. (2019). Decreased maternal serum acetate and impaired fetal thymic and regulatory T cell development in preeclampsia. Nat. Commun..

[26] Munger K.L., Åivo J., Hongell K., Soilu-Hänninen M., Surcel H.-M., Ascherio A. (2016). Vitamin D Status during Pregnancy and Risk of Multiple Sclerosis in Offspring of Women in the Finnish Maternity Cohort. JAMA Neurol..

[27] Nielsen N.M., Corn G., Frisch M., Stenager E., Koch-Henriksen N., Wohlfahrt J., Magyari M., Melbye M. (2019). Multiple sclerosis among first- and second-generation immigrants in Denmark: A population-based cohort study. Brain.

[28] Hawkes C.H., Giovannoni G., Lechner-Scott J., Levy M., Waubant E. (2019). Multiple sclerosis and migration revisited. Mult. Scler. Relat. Disord..

[29] Barnett M.H., McLeod J.G., Hammond S.R., Kurtzke J.F. (2016). Migration and multiple sclerosis in immigrants from United Kingdom and Ireland to Australia: A reassessment. III: Risk of multiple sclerosis in UKI immigrants and Australian-born in Hobart, Tasmania. J. Neurol..

[30] Dean G., Elian M. (1997). Age at immigration to England of Asian and Caribbean immigrants and the risk of developing multiple sclerosis. J. Neurol. Neurosurg. Psychiatry.

[31] Berg-Hansen P., Celius E.G. (2015). Socio-economic factors and immigrant population studies of multiple sclerosis. Acta Neurol. Scand..

[32] Fung T.C., Olson C.A., Hsiao E.Y. (2017). Interactions between the microbiota, immune and nervous systems in health and disease. Nat. Neurosci..

[33] Tanaka M., Nakayama J. (2017). Development of the gut microbiota in infancy and its impact on health in later life. Allergol. Int..

[34] Laforest-Lapointe I., Arrieta M.-C. (2017). Patterns of Early-Life Gut Microbial Colonization during Human Immune Development: An Ecological Perspective. Front. Immunol..

[35] Arrieta M.-C., Stiemsma L.T., Dimitriu P.A., Thorson L., Russell S., Yurist-Doutsch S., Kuzeljevic B., Gold M.J., Britton H.M., Lefebvre D.L. (2015). Early infancy microbial and metabolic alterations affect risk of childhood asthma. Sci. Transl. Med..

[36] Vatanen T., Kostic A.D., d’Hennezel E., Siljander H., Franzosa E.A., Yassour M., Kolde R., Vlamakis H., Arthur T.D., Hamalainen A.M. (2016). Variation in Microbiome LPS Immunogenicity Contributes to Autoimmunity in Humans. Cell.

[37] Haghikia A., Jörg S., Duscha A., Berg J., Manzel A., Waschbisch A., Hammer A., Lee D.H., May C., Wilck N. (2015). Dietary Fatty Acids Directly Impact Central Nervous System Autoimmunity via the Small Intestine. Immunity.

[38] Dopkins N., Nagarkatti P.S., Nagarkatti M. (2018). The role of gut microbiome and associated metabolome in the regulation of neuroinflammation in multiple sclerosis and its implications in attenuating chronic inflammation in other inflammatory and autoimmune disorders. Immunology.

[39] Ratajczak W., Rył A., Mizerski A., Walczakiewicz K., Sipak O., Laszczyńska M. (2019). Immunomodulatory potential of gut microbiome-derived short-chain fatty acids (SCFAs). Acta Biochim. Pol..

[40] Silva Y.P., Bernardi A., Frozza R.L. (2020). The Role of Short-Chain Fatty Acids from Gut Microbiota in Gut-Brain Communication. Front. Endocrinol..

[41] Saresella M., Marventano I., Barone M., La Rosa F., Piancone F., Mendozzi L., d’Arma A., Rossi V., Pugnetti L., Roda G. (2020). Alterations in Circulating Fatty Acid Are Associated With Gut Microbiota Dysbiosis and Inflammation in Multiple Sclerosis. Front. Immunol..

[42] Luu M., Pautz S., Kohl V., Singh R., Romero R., Lucas S., Hofmann J., Raifer H., Vacharajani N., Carrascosa L.C. (2019). The short-chain fatty acid pentanoate suppresses autoimmunity by modulating the metabolic-epigenetic crosstalk in lymphocytes. Nat. Commun..

[43] Dong Y., Yong V.W. (2019). When encephalitogenic T cells collaborate with microglia in multiple sclerosis. Nat. Rev. Neurol..

[44] Barroso A., Mahler J.V., Fonseca-Castro P.H., Quintana F.J. (2021). The aryl hydrocarbon receptor and the gut–brain axis. Cell Mol. Immunol..

[45] Gaetani L., Boscaro F., Pieraccini G., Calabresi P., Romani L., Di Filippo M., Zelante T. (2020). Host and Microbial Tryptophan Metabolic Profiling in Multiple Sclerosis. Front. Immunol..

[46] Mangalam A., Poisson L., Nemutlu E., Datta I., Denic A., Dzeja P., Rodriguez M., Rattan R., Giri S. (2013). Profile of Circulatory Metabolites in an Animal Model of Multiple Sclerosis using Global Metabolomics. J. Clin. Cell Immunol..

[47] Rothhammer V., Borucki D.M., Tjon E.C., Takenaka M.C., Chao C.-C., Ardura-Fabregat A., de Lima K.A., Vazquez C.G., Hewson P., Staszewski O. (2018). Microglial control of astrocytes in response to microbial metabolites. Nature.

[48] Lim C.K., Bilgin A., Lovejoy D.B., Tan V., Bustamante S., Taylor B.V., Bessede A., Brew B.J., Guillemin G.J. (2017). Kynurenine pathway metabolomics predicts and provides mechanistic insight into multiple sclerosis progression. Sci. Rep..

[49] Erny D., Hrabě de Angelis A.L., Jaitin D., Wieghofer P., Staszewski O., David E., Keren-Shaul H., Mahlakoiv T., Jakobshagen K., Buch T. (2015). Host microbiota constantly control maturation and function of microglia in the CNS. Nat. Neurosci..

[50] Duscha A., Gisevius B., Hirschberg S., Yissachar N., Stangl G.I., Eilers E., Bader V., Haase S., Kaisler J., David C. (2020). Propionic Acid Shapes the Multiple Sclerosis Disease Course by an Immunomodulatory Mechanism. Cell.

[51] Cosorich I., Dalla-Costa G., Sorini C., Ferrarese R., Messina M.J., Dolpady J., Radice E., Mariani A., Testoni P.A., Canducci F. (2017). High frequency of intestinal T _H_ 17 cells correlates with microbiota alterations and disease activity in multiple sclerosis. Sci. Adv..

[52] Choileáin S.N., Kleinewietfeld M., Raddassi K., Hafler D.A., Ruff W.E., Longbrake E.E. (2020). CXCR3+ T cells in multiple sclerosis correlate with reduced diversity of the gut microbiome. J. Transl. Autoimmun..

[53] Duc D., Vigne S., Bernier-Latmani J., Yersin Y., Ruiz F., Gaïa N., Leo S., Lazarevic V., Schrenzel J., Petrova T.V. (2019). Disrupting Myelin-Specific Th17 Cell Gut Homing Confers Protection in an Adoptive Transfer Experimental Autoimmune Encephalomyelitis. Cell Rep..

[54] Berer K., Boziki M., Krishnamoorthy G. (2014). Selective Accumulation of Pro-Inflammatory T Cells in the Intestine Contributes to the Resistance to Autoimmune Demyelinating Disease. PLoS ONE.

[55] Haupeltshofer S., Leichsenring T., Berg S., Pedreiturria X., Joachim S.C., Tischoff I., Otte J.M., Bopp T., Fantini M.C., Esser C. (2019). Smad7 in intestinal CD4+ T cells determines autoimmunity in a spontaneous model of multiple sclerosis. Proc. Natl. Acad. Sci. USA.

[56] Gandy K.A.O., Zhang J., Nagarkatti P., Nagarkatti M. (2019). The role of gut microbiota in shaping the relapse-remitting and chronic-progressive forms of multiple sclerosis in mouse models. Sci. Rep..

[57] Held K., Bhonsle-Deeng L., Siewert K., Sato W., Beltrán E., Schmidt S., Ruhl G., Ng J.K.M., Engerer P., Moser M. (2015). αβ T-cell receptors from multiple sclerosis brain lesions show MAIT cell–related features. Neurol. Neuroimmunol. Neuroinflamm..

[58] Carnero Contentti E., Farez M.F., Correale J. (2019). Mucosal-Associated Invariant T cell Features and TCR Repertoire Characteristics during the Course of Multiple Sclerosis. Front. Immunol..

[59] Reboldi A., Cyster J.G. (2016). Peyer’s patches: Organizing B-cell responses at the intestinal frontier. Immunol. Rev..

[60] Rojas O.L., Pröbstel A.-K., Porfilio E.A., Wang A.A., Charabati M., Sun T., Lee D.S.W., Galicia G., Ramaglia V., Ward L.A. (2019). Recirculating Intestinal IgA-Producing Cells Regulate Neuroinflammation via IL-10. Cell.

[61] Planas R., Santos R., Tomas-Ojer P., Cruciani C., Lutterotti A., Faigle W., Schaeren-Wiemers N., Espejo C., Eixarch E., Pinilla C. (2018). GDP-l-fucose synthase is a CD4 ^+^ T cell–specific autoantigen in DRB3*02, 02 patients with multiple sclerosis. Sci. Transl. Med..

[62] Mecha M., Carrillo-Salinas F.J., Mestre L., Feliú A., Guaza C. (2013). Viral models of multiple sclerosis: Neurodegeneration and demyelination in mice infected with Theiler’s virus. Prog. Neurobiol..

[63] Küry P., Nath A., Créange A., Dolei A., Marche P., Gold J., Giovannoni G., Hartung H.P., Perron H. (2018). Human Endogenous Retroviruses in Neurological Diseases. Trends Mol. Med..

[64] Perron H., Garson J.A., Bedin F., Beseme F., Paranhos-Baccala G., Komurian-Pradel F., Mallet F., Tuke P.W., Voisset C., Blond J.L. (1997). Molecular identification of a novel retrovirus repeatedly isolated from patients with multiple sclerosis. Proc. Natl. Acad. Sci. USA.

[65] Morandi E., Tanasescu R., Tarlinton R.E., Constantinescu C.S., Zhang W., Tench C., Gran B. (2017). The association between human endogenous retroviruses and multiple sclerosis: A systematic review and meta-analysis. PLoS ONE.

[66] Handel A.E., Williamson A.J., Disanto G., Handunnetthi L., Giovannoni G., Ramagopalan S.V. (2010). An Updated Meta-Analysis of Risk of Multiple Sclerosis following Infectious Mononucleosis. PLoS ONE.

[67] Afrasiabi A., Parnell G.P., Swaminathan S., Stewart G.J., Booth D.R. (2020). The interaction of Multiple Sclerosis risk loci with Epstein-Barr virus phenotypes implicates the virus in pathogenesis. Sci. Rep..

[68] Pakpoor J., Disanto G., Gerber J.E., Dobson R., Meier U.C., Giovannoni G., Ramagopalan S.V. (2013). The risk of developing multiple sclerosis in individuals seronegative for Epstein-Barr virus: A meta-analysis. Mult. Scler. J..

[69] Abrahamyan S., Eberspächer B., Hoshi M.-M., Aly L., Luessi F., Groppa S., Klotz L., Meuth S.G., Schroeder C., Gruter T. (2020). Complete Epstein-Barr virus seropositivity in a large cohort of patients with early multiple sclerosis. J. Neurol. Neurosurg. Psychiatry.

[70] Libbey J.E., McCoy L.L., Fujinami R.S. (2007). Molecular Mimicry in Multiple Sclerosis. International Review of Neurobiology.

[71] Croxford J.L., Olson J.K., Anger H.A., Miller S.D. (2005). Initiation and Exacerbation of Autoimmune Demyelination of the Central Nervous System via Virus-Induced Molecular Mimicry: Implications for the Pathogenesis of Multiple Sclerosis. J. Virol..

[72] Jog N.R., McClain M.T., Heinlen L.D., Gross T., Towner R., Guthridge J.M., Axtell R.C., Pardo G., Harley J.B., James J.A. (2020). Epstein Barr virus nuclear antigen 1 (EBNA-1) peptides recognized by adult multiple sclerosis patient sera induce neurologic symptoms in a murine model. J. Autoimmun..

[73] Tengvall K., Huang J., Hellström C., Kammer P., Biström M., Ayoglu B., Bomfim I.L., Stridh P., Butt J., Brenner N. (2019). Molecular mimicry between Anoctamin 2 and Epstein-Barr virus nuclear antigen 1 associates with multiple sclerosis risk. Proc. Natl. Acad. Sci. USA.

[74] Tao C., Simpson S., Taylor B.V., van der Mei I. (2017). Association between human herpesvirus & human endogenous retrovirus and MS onset & progression. J. Neurol. Sci..

[75] Opsahl M.L., Kennedy P.G.E. (2005). Early and late HHV-6 gene transcripts in multiple sclerosis lesions and normal appearing white matter. Brain.

[76] Engdahl E., Gustafsson R., Huang J., Biström M., Lima Bomfim I., Stridh P., Khademi M., Brenner N., Butt J., Michel A. (2019). Increased Serological Response Against Human Herpesvirus 6A Is Associated with Risk for Multiple Sclerosis. Front. Immunol..

[77] Ahlqvist J., Fotheringham J., Akhyani N., Yao K., Fogdell-Hahn A., Jacobson S. (2005). Differential tropism of human herpesvirus 6 (HHV-6) variants and induction of latency by HHV-6A in oligodendrocytes. J. Neurovirol..

[78] Kremer D., Gruchot J., Weyers V., Oldemeier L., Göttle P., Healy L., Jang J.H., Xu Y.K.T., Volsko C., Dutta R. (2019). pHERV-W envelope protein fuels microglial cell-dependent damage of myelinated axons in multiple sclerosis. Proc. Natl. Acad. Sci. USA.

[79] GeNeuro S.A. (2020). An International, Double-blind, Randomised, Placebo-controlled Phase IIb Trial to Assess the Efficacy, Safety, and Pharmacokinetics of GNbAC1 in Patients with Relapsing Remitting Multiple Sclerosis. NCT02782858.

[80] Mazzoni E., Bononi I., Pietrobon S., Torreggiani E., Rossini M., Pugliatti M., Casetta I., Castellazzi M., Granieri E., Guerra G. (2020). Specific antibodies reacting to JC polyomavirus capsid protein mimotopes in sera from multiple sclerosis and other neurological diseases-affected patients. J. Cell Physiol..

[81] Mazzoni E., Pietrobon S., Masini I., Rotondo J.C., Gentile M., Fainardi E., Casetta I., Castellazzi M., Granieri E., Caniati M.L. (2014). Significant low prevalence of antibodies reacting with simian virus 40 mimotopes in serum samples from patients affected by inflammatory neurologic diseases, including multiple sclerosis. PLoS ONE.

[82] Rizzo R., Pietrobon S., Mazzoni E., Bortolotti D., Martini F., Castellazzi M., Cassetta I., Fainardi E., Di Luca D., Granieri E. (2016). Serum IgG against Simian Virus 40 antigens are hampered by high levels of sHLA-G in patients affected by inflammatory neurological diseases, as multiple sclerosis. J. Transl. Med..

[83] Carter C.J. (2012). Epstein–Barr and other viral mimicry of autoantigens, myelin and vitamin D-related proteins and of EIF2B, the cause of vanishing white matter disease: Massive mimicry of multiple sclerosis relevant proteins by the *Synechococcus* phage. Immunopharmacol. Immunotoxicol..

[84] Tetz G., Tetz V. (2016). Bacteriophage infections of microbiota can lead to leaky gut in an experimental rodent model. Gut Pathog..

[85] Tetz G.V., Ruggles K.V., Zhou H., Heguy A., Tsirigos A., Tetz V. (2017). Bacteriophages as potential new mammalian pathogens. Sci. Rep..

[86] Zhao G., Vatanen T., Droit L., Park A., Kostic A.D., Poon T.W., Vlamakis H., Siljander H., Harkonen T., Hamalainen A.-M. (2017). Intestinal virome changes precede autoimmunity in type I diabetes-susceptible children. Proc. Natl. Acad. Sci. USA.

[87] Kumata R., Ito J., Takahashi K., Suzuki T., Sato K. (2020). A tissue level atlas of the healthy human virome. BMC Biol..

[88] Guo C.-J., Chang F.-Y., Wyche T.P., Backus K.M., Acker T.M., Funabashi M., Taketani M., Donia M.S., Nayfach S., Pollard K.S. (2017). Discovery of Reactive Microbiota-Derived Metabolites that Inhibit Host Proteases. Cell.

[89] Vincents B., Vindebro R., Abrahamson M., von Pawel-Rammingen U. (2008). The Human Protease Inhibitor Cystatin C Is an Activating Cofactor for the Streptococcal Cysteine Protease IdeS. Chem. Biol..

[90] Hoghooghi V., Palmer A.L., Frederick A., Jiang Y., Merkens J.E., Balakrishnan A., Finlay T.M., Grubb A., Levy E., Gordon P. (2020). Cystatin C Plays a Sex-Dependent Detrimental Role in Experimental Autoimmune Encephalomyelitis. Cell Rep..

[91] Gonzalez C.G., Tankou S.K., Cox L.M., Casavant E.P., Weiner H.L., Elias J.E. (2019). Latent-period stool proteomic assay of multiple sclerosis model indicates protective capacity of host-expressed protease inhibitors. Sci. Rep..

[92] Miyake S., Kim S., Suda W., Oshima K., Nakamura M., Matsuoka T., Chihara N., Tomita A., Sato W., Kim S.-W. (2015). Dysbiosis in the Gut Microbiota of Patients with Multiple Sclerosis, with a Striking Depletion of Species Belonging to Clostridia XIVa and IV Clusters. PLoS ONE.

[93] Huynh J.L., Garg P., Thin T.H., Yoo S., Dutta R., Trapp B.D., Haroutunian V., Zhu J., Donovan M.J., Sharp A.J. (2014). Epigenome-wide differences in pathology-free regions of multiple sclerosis–affected brains. Nat. Neurosci..

[94] Allan E.R.O., Campden R.I., Ewanchuk B.W., Tailor P., Balce D.R., McKenna N.T., Greene C.J., Warren A.L., Reinheckel T., Yates R.M. (2017). A role for cathepsin Z in neuroinflammation provides mechanistic support for an epigenetic risk factor in multiple sclerosis. J. Neuroinflammation.

[95] Arpaia N., Campbell C., Fan X., Dikiy S., van der Veeken J., de Roos P., Liu H., Cross J.R., Pfeffer K., Coffer P.J. (2013). Metabolites produced by commensal bacteria promote peripheral regulatory T-cell generation. Nature.

[96] Atarashi K., Tanoue T., Shima T., Imaoka A., Kuwahara T., Momose Y., Cheng G., Yamasaki S., Saito T., Ohba Y. (2011). Induction of Colonic Regulatory T Cells by Indigenous Clostridium Species. Science.

[97] Mizuno M., Noto D., Kaga N., Chiba A., Miyake S. (2017). The dual role of short fatty acid chains in the pathogenesis of autoimmune disease models. PLoS ONE.

[98] Gödel C., Kunkel B., Kashani A., Lassmann H., Arumugam M., Krishnamoorthy G. (2020). Perturbation of gut microbiota decreases susceptibility but does not modulate ongoing autoimmune neurological disease. J. Neuroinflammation.

[99] Lee Y.K., Menezes J.S., Umesaki Y., Mazmanian S.K. (2011). Proinflammatory T-cell responses to gut microbiota promote experimental autoimmune encephalomyelitis. Proc. Natl. Acad. Sci. USA.

[100] Belkaid Y., Hand T.W. (2014). Role of the Microbiota in Immunity and Inflammation. Cell.

[101] McMurran C.E., Guzman de la Fuente A., Penalva R., Ben Menachem-Zidon O., Dombrowski Y., Falconer J., Gonzalez G.A., Zhao C., Krause F.N., Young A.M.H. (2019). The microbiota regulates murine inflammatory responses to toxin-induced CNS demyelination but has minimal impact on remyelination. Proc. Natl. Acad. Sci. USA.

[102] Mager L.F., Burkhard R., Pett N., Cooke N.C.A., Brown K., Ramay H., Paik S., Stagg J., Groves R.A., Gallo M. (2020). Microbiome-derived inosine modulates response to checkpoint inhibitor immunotherapy. Science.

[103] Shahi S.K., Freedman S.N., Murra A.C., Zarei K., Sompallae R., Gibson-Corley K.N., Karandikar N.J., Murray J.A., Mangalam A.K. (2019). Prevotella histicola, A Human Gut Commensal, Is as Potent as COPAXONE^®^ in an Animal Model of Multiple Sclerosis. Front. Immunol..

[104] Odoardi F., Sie C., Streyl K., Ulaganathan V.K., Schläger C., Lodygin D., Heckelsmiller K., Nietfeld W., Ellwart J., Klinkert W.E.F. (2012). T cells become licensed in the lung to enter the central nervous system. Nature.

[105] Hassani Y., Brégeon F., Aboudharam G., Drancourt M., Grine G. (2020). Detection of Methanobrevobacter smithii and Methanobrevibacter oralis in Lower Respiratory Tract Microbiota. Microorganisms.

[106] Koskinen K., Pausan M.R., Perras A.K., Beck M., Bang C., Mora M., Schilhabel A., Schmitz R., Moissl-Eichinger C. (2017). First Insights into the Diverse Human Archaeome: Specific Detection of Archaea in the Gastrointestinal Tract, Lung, and Nose and on Skin. mBio.

[107] Dixit A., Tanaka A., Greer J.M., Donnelly S. (2017). Novel Therapeutics for Multiple Sclerosis Designed by Parasitic Worms. IJMS.

[108] Tanasescu R., Tench C.R., Constantinescu C.S., Telford G., Singh S., Frakich N., Onion D., Auer D.P., Gran B., Evangelou N. (2020). Hookworm Treatment for Relapsing Multiple Sclerosis: A Randomized Double-Blinded Placebo-Controlled Trial. JAMA Neurol..

[109] Lund M.E., Greer J., Dixit A., Alvarado R., McCauley-Winter P., To J., Tanaka A., Hutchinson A.T., Robinson M.W., Simpson A.M. (2016). A parasite-derived 68-mer peptide ameliorates autoimmune disease in murine models of Type 1 diabetes and multiple sclerosis. Sci. Rep..

[110] Marzano V., Mancinelli L., Bracaglia G., Del Chierico F., Vernocchi P., Di Girolamo F., Garrone S., Kuekou H.T., D’Argenio P., Dallapiccola B. (2017). “Omic” investigations of protozoa and worms for a deeper understanding of the human gut “parasitome”. PLoS Negl. Trop. Dis..

[111] Lozinski B.M., Yong V.W. (2020). Exercise and the brain in multiple sclerosis. Mult. Scler..

[112] Nieman D.C., Wentz L.M. (2019). The compelling link between physical activity and the body’s defense system. J. Sport Health Sci..

[113] Bermon S., Petriz B., Kajėnienė A., Prestes J., Castell L., Franco O.L. (2015). The microbiota: An exercise immunology perspective. Exerc. Immunol. Rev..

[114] Estaki M., Pither J., Baumeister P., Little J.P., Gill S.K., Ghosh S., Ahmadi-Vand Z., Marsden K.R., Gibson D.L. (2016). Cardiorespiratory fitness as a predictor of intestinal microbial diversity and distinct metagenomic functions. Microbiome.

[115] Campbell S.C., Wisniewski P.J., Noji M., McGuinness L.R., Häggblom M.M., Lightfoot S.A., Joseph L.B., Kerkhof L.J. (2016). The Effect of Diet and Exercise on Intestinal Integrity and Microbial Diversity in Mice. PLoS ONE.

[116] Barton W., Penney N.C., Cronin O., Garcia-Perez I., Molloy M.G., Holmes E., Shanahan F., Cotter P.D., O’Sullivan O. (2018). The microbiome of professional athletes differs from that of more sedentary subjects in composition and particularly at the functional metabolic level. Gut.

[117] Codella R., Luzi L., Terruzzi I. (2018). Exercise has the guts: How physical activity may positively modulate gut microbiota in chronic and immune-based diseases. Dig. Liver Dis..

[118] Quiroga R., Nistal E., Estébanez B., Porras D., Juárez-Fernández M., Martínez-Flórez S., Garcia-Mediavilla M.V., de Paz J.A., Gonzalez-Gallego J., Sanchez-Campos S. (2020). Exercise training modulates the gut microbiota profile and impairs inflammatory signaling pathways in obese children. Exp. Mol. Med..

[119] Mitchell C.M., Davy B.M., Hulver M.W., Neilson A.P., Bennett B.J., Davy K.P. (2019). Does Exercise Alter Gut Microbial Composition?. A Syst. Rev. Med. Sci. Sports Exerc..

[120] Liu Y., Wang Y., Ni Y., Cheung C.K.Y., Lam K.S.L., Wang Y., Xia Z., Ye D., Guo J., Tse M.A. (2020). Gut Microbiome Fermentation Determines the Efficacy of Exercise for Diabetes Prevention. Cell Metab..

[121] Bressa C., Bailén-Andrino M., Pérez-Santiago J., González-Soltero R., Pérez M., Montalvo-Lominchar M.G., Mate-Munoz J.L., Dominguez R., Moreno D., Larrosa M. (2017). Differences in gut microbiota profile between women with active lifestyle and sedentary women. PLoS ONE.

[122] Jensen S.K., Michaels N.J., Ilyntskyy S., Keough M.B., Kovalchuk O., Yong V.W. (2018). Multimodal Enhancement of Remyelination by Exercise with a Pivotal Role for Oligodendroglial PGC1α. Cell Rep..

[123] Magyari M., Sorensen P.S. (2020). Comorbidity in Multiple Sclerosis. Front Neurol..

[124] Perumal J., Fox R.J., Balabanov R., Balcer L.J., Galetta S., Makh S., Santra S., Hotermans C., Lee L. (2019). Outcomes of natalizumab treatment within 3 years of relapsing-remitting multiple sclerosis diagnosis: A prespecified 2-year interim analysis of STRIVE. BMC Neurol..

[125] Butzkueven H., Kappos L., Wiendl H., Trojano M., Spelman T., Chang I., Kasliwal R., Jaitly S., Campell N., Ho P.-R. (2020). Long-term safety and effectiveness of natalizumab treatment in clinical practice: 10 years of real-world data from the Tysabri Observational Program (TOP). J. Neurol. Neurosurg. Psychiatry.

[126] Brandstadter R., Katz Sand I. (2017). The use of natalizumab for multiple sclerosis. NDT.

[127] Wang H., Lee I.-S., Braun C., Enck P. (2016). Effect of Probiotics on Central Nervous System Functions in Animals and Humans: A Systematic Review. J. Neurogastroenterol. Motil..

[128] Feinstein A., Magalhaes S., Richard J.-F., Audet B., Moore C. (2014). The link between multiple sclerosis and depression. Nat. Rev. Neurol..

[129] Jakimovski D., Guan Y., Ramanathan M., Weinstock-Guttman B., Zivadinov R. (2019). Lifestyle-based modifiable risk factors in multiple sclerosis: Review of experimental and clinical findings. Neurodegener. Dis. Manag..

[130] Haran J.P., Bucci V., Dutta P., Ward D., McCormick B. (2018). The nursing home elder microbiome stability and associations with age, frailty, nutrition and physical location. J. Med Microbiol..

[131] Murphy R., Harrison J., Schelenz S., Davies J. (2019). M5 The multiple sclerosis drug, glatiramer acetate, acts as a resistance breaker with antibiotics from different classes against cystic fibrosis strains of pseudomonas aeruginosa. The Epidemiology and Impact of Difficult Infections.

[132] Metz L.M., Li D.K.B., Traboulsee A.L., Duquette P., Eliasziw M., Cerchiaro G., Greenfield J., Riddehough A., Yeung M., Kremenchutzky M. (2017). Trial of Minocycline in a Clinically Isolated Syndrome of Multiple Sclerosis. N. Engl. J. Med..

[133] Jia L., Zhang M., Liu H., Sun J., Pan L. (2021). Early-life fingolimod treatment improves intestinal homeostasis and pancreatic immune tolerance in non-obese diabetic mice. Acta Pharmacol. Sin..

[134] Rumah K.R., Vartanian T.K., Fischetti V.A. (2017). Oral Multiple Sclerosis Drugs Inhibit the In vitro Growth of Epsilon Toxin Producing Gut Bacterium, Clostridium perfringens. Front. Cell Infect. Microbiol..

